# Extracellular Vesicles and the Inflammasome: An Intricate Network Sustaining Chemoresistance

**DOI:** 10.3389/fonc.2022.888135

**Published:** 2022-04-22

**Authors:** Letizia Mezzasoma, Ilaria Bellezza, Rita Romani, Vincenzo Nicola Talesa

**Affiliations:** Department of Medicine and Surgery, University of Perugia, Perugia, Italy

**Keywords:** extracellular vesicles, inflammasome, tumor microenvironment, IL-1β, chemoresistance

## Abstract

Extracellular vesicles (EVs) are membrane enclosed spherical particles devoted to intercellular communication. Cancer-derived EVs (Ca-EVs) are deeply involved in tumor microenvironment remodeling, modifying the inflammatory phenotype of cancerous and non-cancerous residing cells. Inflammation plays a pivotal role in initiation, development, and progression of many types of malignancies. The key feature of cancer-related inflammation is the production of cytokines that incessantly modify of the surrounding environment. Interleukin-1β (IL-1β) is one of the most powerful cytokines, influencing all the initiation-to-progression stages of many types of cancers and represents an emerging critical contributor to chemoresistance. IL-1β production strictly depends on the activation of inflammasome, a cytoplasmic molecular platform sensing exogenous and endogenous danger signals. It has been recently shown that Ca-EVs can activate the inflammasome cascade and IL-1β production in tumor microenvironment-residing cells. Since inflammasome dysregulation has been established as crucial regulator in inflammation-associated tumorigenesis and chemoresistance, it is conceivable that the use of inflammasome-inhibiting drugs may be employed as adjuvant chemotherapy to counteract chemoresistance. This review focuses on the role of cancer-derived EVs in tuning tumor microenvironment unveiling the intricate network between inflammasome and chemoresistance.

## Introduction

In the complexity of cancer progression, the intricate interplay between inflammation and tumor microenvironment (TME), depicts an extraordinary multifaceted scenario in the development of acquired drug resistance and in the clinical outcome of malignant processes (1, 2). Extracellular vesicles (EVs), in particular cancer-derived (Ca-EVs), represent signal transducer or messengers in cell-cell communication (3–5), responsible for the continuous modification of TME (6, 7). TME includes cancerous and non-cancerous cellular components such as fibroblasts, stromal, immune, and endothelial cells. The cross-talk between TME components can induce a dysregulated inflammatory and immune response (1, 2). Inflammation, indeed, plays a pivotal role in tumor initiation, by dynamically and incessantly modifying TME *via* the release of cytokines and soluble mediators generating a “vicious cycle”. This, in turn, endorses oncogenic plasticity toward immune-suppression, more aggressive phenotype and reduction of therapeutic efficacy. One of the main mechanisms contributing to inflammation is mediated by cytoplasmatic complexes known as inflammasomes. Inflammasomes are activated by endogenous/exogenous danger signals and changes in cytoplasm homeostasis. Upon activation, inflammasomes act as “signal integrators” by the release of inflammasome-effectors cytokines. Inflammasomes are pivotal hubs of innate immunity and modulate immune/inflammatory responses by cross-talking with different cellular components. Inflammasome inappropriate activation, creating a pro-inflammatory TME and suppressing local immunity, appears as an emerging player in all the initiation to progression stages of cancer (8–11). Crucial novel modulators of inflammasome are EVs that, on the basis of the different nature of their cellular source, positively or negatively affect inflammasome cascade in diverse cancerous and non-cancerous recipient cells (12–14). In this scenario, EVs, with their Janus face behavior, strongly contribute to the immune/inflammation-associated modification of TME, and play a critical role in tumorigenesis and chemoresistance.

## EVs: Another Brick in the Wall

EVs are a heterogeneous group of membrane enwrapped spherical particles, produced by nearly all types of cells. There are no unique markers able to classify EVs on the bases on their biogenesis (ectosomes, exosomes, apoptotic bodies), for this reason the MISEV2018 guidelines suggest classifying EVs based on physical parameters, such as size (small and medium/large EVs) density or biochemical composition (15–17). EVs, found in body fluids and in cell culture media, carry various biomolecules, including proteins, lipids, metabolites, RNA, and DNA (16, 18). Upon interaction with target cell, EVs deeply impact cellular recipient cells responses, highlighting the pivotal role of EVs as signal transducers or messengers in cell-cell communication at close or distant sites. Intercellular communication is a key feature of tumor progression and metastasis. Cancer cells can release EVs that enter the circulation and reach distant organs, where they can generate favorable environmental conditions, enabling the outgrowth of disseminated tumor cells. This process, known as pre-metastatic niche formation, requires a series of predefined steps involving induction of vascular leakiness, alteration of stromal components and immune-escape (19, 20).

Cancer-derived EVs (Ca-EVs) ability to suppress immune anti-tumor activity, is guaranteed by the exchange of EVs between cancerous and non-cancerous TME-residing cells, and by the secretion of immune-modulating molecules (14, 21). Furthermore, the “exosome-immune suppression” and the Ca-EVs-mediated transfer of oncogenes or oncometabolites from one cell to other is also involved in the unrestrained cell proliferation and, subsequently, in the metastatic spread (14). On the other hand, Ca-EVs, may also carry tumor-associated antigens, damage associated molecular pattern (DAMPs), and immune-stimulating molecules, that can induce an immune anti-tumor response (22, 23) *via* the recruitment and activation of immune cells in TME (24, 25). Although the pro-inflammatory and the immune-suppressive role of Ca-EVs seem to be contrasting, pro-inflammatory EVs may still contribute to TME maintenance (26, 27).

## Interleukin-1β and Chemoresistance: An Old Cytokine With a Novel Role

Interleukin-1β (IL-1β) is one of the most abundant and influential cytokines of TME. IL-1β expression and secretion are induced by different stimuli such as toll-like receptors (TLRs) ligands, tumor necrosis factor-α or IL-1β itself. IL-1β production/secretion are fine-tune controlled by a two-steps transcriptional and post-translational regulation, requiring the activation of both nuclear factor kappa B (NF-κB) and nucleotide-binding oligomerization domain (NOD)-like receptor pyrin domain-containing 3 (NLRP3) inflammasome-caspase-1 platform. NF-κB activation by inflammatory stimuli induces biologically inactive pro-IL-1β production which must be proteolytically cleaved, by inflammasome-activated caspase-1 (28). Tumor cells can directly produce IL-1β or can “instruct” cells within TME, such as stromal ones, to secrete it (26, 29). An uncontrolled increase in IL-1β release exerts immune-suppressive effects and influences all the initiation-to-progression stages of many types of cancers and represents an emerging critical contributor to chemoresistance (30, 31). Depending on tumor cell types, several “*in vitro*” and “*in vivo*” models highlighted multiple mechanisms for IL-1β promoted chemoresistance. In pleural mesothelioma, the IL-8/IL-1β signaling controls chemoresistance by inducing the overexpression of ATP-binding cassette transporter (ABC) G2, that determines resistance to cisplatin and pemetrexed (32). Prostate carcinoma cells engage bone marrow adipocytes in a functional cyclooxygenase-2 (COX-2)-dependent cross-talk that promotes IL-1β expression, leading to docetaxel resistance (33). IL-1β can also induce a reinforcement of NF-κB signaling. In fact, IL-1β induces a sustained NF-κB that has been related to chemoresistance in ovarian carcinoma (34), in acute myeloid leukaemia (35) and in renal cell carcinoma (36). In pancreatic cancer, IL-1β confers chemoresistance not only by activating NF-κB (37), but also by up-regulating COX-2 (38), an enzyme linked to chemoresistance also in cervical carcinoma (39) and in colon cancer cell lines (40). In bladder cancer, cisplatin-resistance has been linked to IL-1β-induced increase in aldo-keto reductase 1C1 levels (41). In breast cancer, IL-1β-induced chemoresistance has been attributed to several mechanisms including: methylation of the estrogen receptor α, which increases tamoxifen resistance (42); activation of β-catenin signaling, which increases cisplatin resistance (43); and induction of epithelial to mesenchymal transition (EMT), which increases doxorubicin resistance (44). In melanoma cells, ABCB5 controls IL-1β/IL-8 signaling (45) which, in turn, influences chemoresistance by activating Smad/DNA binding protein 1 signaling (46).

Considering the implication of IL-1β in influencing all the initiation-to-progression stages of many tumors and chemoresistance, this cytokine is considered a promising therapeutic target for many types of cancers (30).

## The Inflammasome: A Double-Edge Sword

As already mentioned, inflammasome activation is the mandatory event for IL-1β maturation and secretion. Inflammasomes are cytoplasmic molecular platforms devoted to detecting pathogen associated molecular patterns (PAMPs) and DAMPs, playing a key role in innate immunity (47). The inflammasome platform is composed by a danger sensor receptor, an adaptor protein (Apoptosis-associated speck-like protein containing a CARD, ASC), and an effector enzyme (caspase-1). The receptor family includes the nucleotide-binding and oligomerization domain (NOD)-like receptors (NLRs) family, composed of at least 22 members, the most characterized of which is NLRP3 (47). Upon activation, NLRP3 oligomerizes and assembles into a multimeric platform including a core unit comprehending ASC and the effector pro-caspase-1. The oligomerization of inflammasome components culminates in the autocatalytic activation of caspase-1, responsible for IL-1β and IL-18 maturation (47–49). Inflammasome activation may also induce the processing of gasdermin-D (GSDMD), leading to pyroptosis, an inflammatory form of cell death (50). The physical interaction among inflammasome components is mediated by the adaptor protein ASC which holds a pyrin (PYD) and a CARD domain which, assembling into a speck, consents the connection between NLRP3 and caspase-1. NLRP3 possesses three domains: an N-terminal effector PYD, involved in ASC recruitment *via* PYD-PYD interaction, a central NACHT domain carrying an ATPase activity essential for NLRP3 activation and platform assembly, and a C-terminal leucine-rich repeats domain, possibly involved in auto-regulation, protein-protein interaction, and signal sensing (51). Inflammasome-platform assembly is also regulated by the phosphorylation of Ser-295 of NLRP3. This post-translational modification, accomplished by several protein kinases (PKs) including PKA, PKD and PKG (52, 53), impedes inflammasome platform assembly. Because component assembly is mandatory for inflammasome activation, it represents an attractive target for the development of selective NLRP3 inhibitors, as discussed later. NLRP3 involvement in cancer is currently a very debated topic. NLRP3 and NLRP3-associated pyroptosis have been defined “a double-edge sword” (54) on the basis of their capability to achieve both an anti-tumorigenic and a pro-tumorigenic activity in different types of malignancies (9–11, 54, 55). The contrasting roles of NLRP3 inflammasome can be due to multiple factors such as type, heterogeneity and stage of cancer cells, or TME characteristics (8–11). Metabolites, cytokines and EVs released by TME residing cells, represent the key drivers of NLRP3 hyper-activation. NLRP3 dysregulated activation can induce a chronic inflammatory environment that boosts tumor progression and extinguishes local immunity (9).

Recently the uncontrolled inflammasome activation has also been associated to chemoresistance. In oral squamous carcinoma NLRP3 activation promotes 5-Fluorouracil resistance “*in vitro*” and “*in vivo*” (56); and NLRP3 inflammasome has been detected in cisplatin-resistant lung cancer cell lines (57). Conversely, NLRP3-induced pyroptosis, sensitizes gastric and epatocellular carcinoma to cisplatin (58, 59).

NLRP3 inflammasome has been also linked to cardiotoxicity of anticancer agents. The inhibition of NLRP3, as well as of the oligomerization of the myeloid differentiation primary response gene 88 (MyD88), reduces the cardiotoxicity and increases the anticancer properties of sunitinib, in renal cancer-bearing mice (60). Myd88 is molecular platform which oligomerization and assembly induces NF-κB activation and the release of cytokine and factors involved in cancer cell survival and chemoresistance (60). Pharmacological reduction of NLRP3 activity has been suggested as a tool to alleviate doxorubicin-induced cardiotoxicity while preserving or even improving its anti-cancer activity (61). On the other hand, pyroptosis‐associated cytokines can induce either an evasion of immune surveillance, or an effective immune response (62).

## EVs and Inflammasome: Two Pieces of the Same Puzzle

Increasing evidence highlights the pivotal role of Ca-EVs on NLRP3 activation in different types of cancers (summarized in **Table 1**). Prostate cancer derived-EVs, by inducing NLRP3 activation and IL-1β maturation, modify the inflammatory response of ME residing cells in a tumor-promoting fashion (12). Furthermore, prostate cancer tumor progression is characterized by increased inflammasome activation (62). Lung cancer-derived EVs induce NLRP3 activation in macrophages, thus providing a positive feedback loop to promote cancer progression *via* IL-1β secretion in mice (63, 64). EVs released by primary cultures of human glioblastoma, up-regulate microglial inflammasome signaling and influence both microglial cells polarization and glioma-microglia crosstalk (65). Furthermore, EVs derived from colon adenocarcinoma cells mediate radiation-induced antitumor immunity by inducing NLRP3 activation in mice (66).

**Table 1:** Roles of EVs and drugs in inflammasome modulation.

	Role and mechanism of action	References
*EVs-mediated inflammasome activation*		
Prostate cancer-derived EVs (PCa-EVs)	PCa-EVs induce caspase-1/IL-1β activation *via* ERK1/2-mediated lysosomal destabilization and cathepsin B activation in non-cancerous PNT2 cells	(12)
Lung cancer-derived EVs (LCa-EVs)	LCa-EVs induce NLRP3-mediated IL-1β secretion in macrophages thus promoting lung cancer development	(63, 64)
Glioblastoma-derived EVs (GMB-EVs)	GMB-EVs induce inflammasome/IL-1β activation in microglial cells thus inducing microglial cells M1 polarization	(65)
Colon adenocarcinoma-derived EVs (CCa-EVs)	CCa-EVs induce AIM2 and NLRP3 activation, and prompt IL-1β-mediated anti-tumor effect during radiation in mice	(66)
*EVs-mediated inflammasome inhibition*		
Amniotic fluid stem cell-derived EVs (HASC-EVs)	HASC-EVs inhibit NLRP3/caspase-1 activation *via* an intrinsic metabolic activity leading to A2a purinergic receptor activation in THP1 cells	(13)
Embryonic stem cells-derived EVs (ES-EVs)	ES-EVs reduce doxorubicin-induced NLRP3/Caspase-1/IL-1β/IL-18/Pyroptosis activation in M1 macrophages thus converting pro-inflammatory M1 into anti-inflammatory M2 macrophages	(67)
*Drug-mediated inflammasome inhibition*		
OLT1177	OLT1177 blocks NLRP3 oligomerization and IL-1β secretion thus enhancing anti-tumor immunity and reducing tumor growth in melanoma cells	(68)
MCC950	MCC950 inhibits NLRP3 activation and reduces tumor growth of pancreatic cancer cells; head and neck squamous adenocarcinoma; and pituitary prolactinoma	(69–71)
Oridonin and its derivate	Oridonin and its derivate impede NLRP3 assembly and prevents liver colorectal cancer metastasis	(72–75)
MM01 and Xantone	MM01 and xantone prevent inflammasome activation interfering with ASC speck formation	(76, 77)
VX-765	VX-765 inhibits caspase-1 activation, thus preventing inflammasome activation and pyroptosis	(78, 79)
Ritonavir	Ritonavir blocks caspase-1 activation in pancreatic cancer	(80, 81)
Anakinra	Anakinra blocks the binding of IL-1 to its receptors. It is under clinical investigation for the treatment of metastatic cancers	(82)
Natriuretic Peptides (NPs)	NPs interfere with NLRP3 activation by the induction of NLRP3 phosphorylation that inhibits ASC oligomerization. NPs counteract inflammasome activation in prostate cancer cell lines	(53, 62, 83, 84)

**Figure 1:**
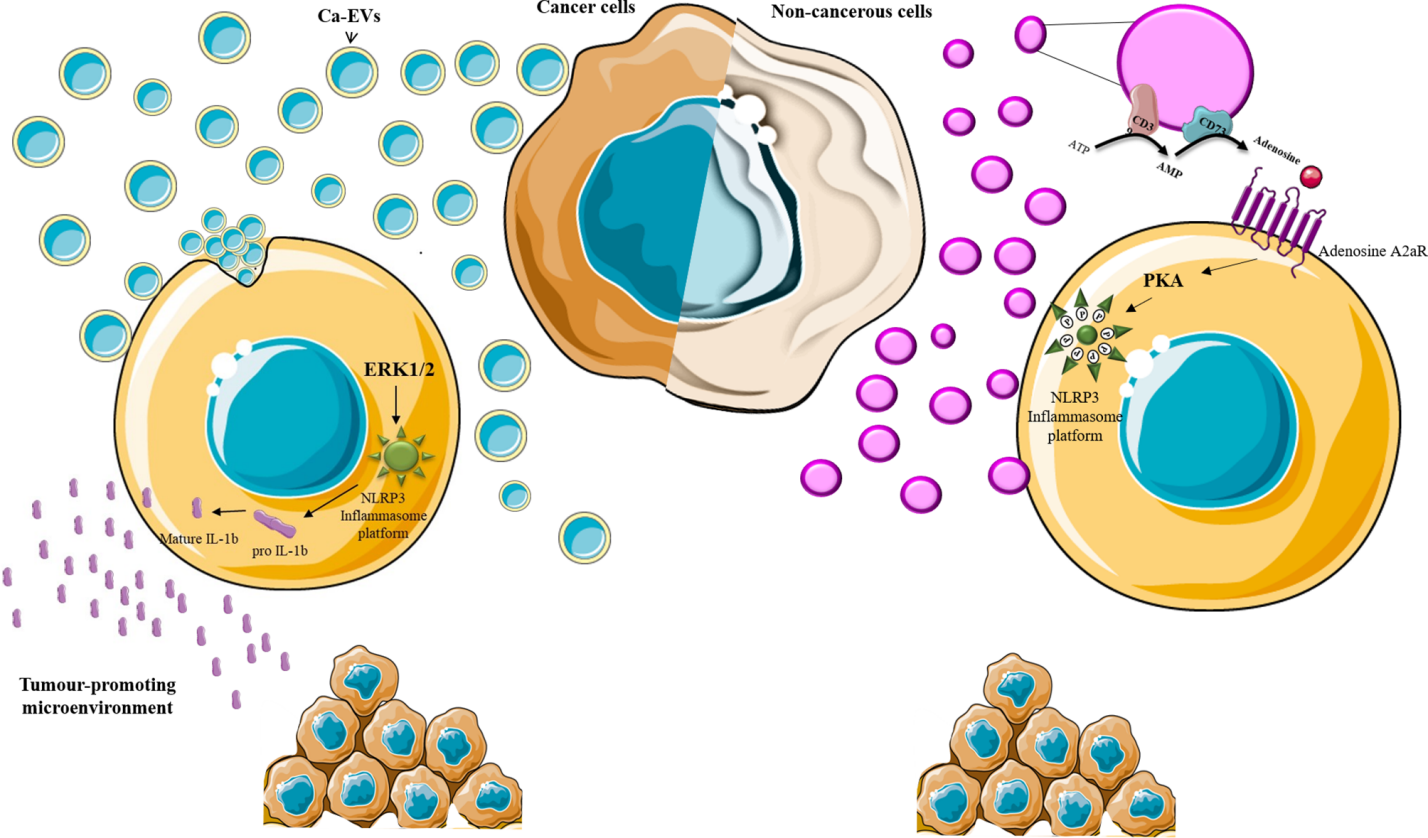
EVs and inflammasome. Schematic representation of EVs-induced effect on inflammasome activation. Ca-EVs (light blue) are up-taken by non-cancerous cells and, *via* the induction of intracellular signaling pathways, including ERK1/2 MAPK, induce inflammasome platform assembly and the maturation of IL-1β which release affects the microenvironment in a tumor-promoting fashion. EVs released by non-cancerous cells violet may produce soluble factor (Adenosine) that, *via* receptor (adenosine A2a receptor) engagement on the target cell, activates protein kinases (PKA) which impedes inflammasome platform assembly through NLRP3 phosphorylation (P).

The role of EVs in immune-escape and immune-stimulation also relies on their ability to modulate inflammasome cascade positively or negatively and IL-1β production in recipient cells (12–14) (summarized in **Table 1**). This different effect is both related to the nature of the EVs-releasing and -receiving cells and to the different EVs mechanisms of action (**Figure 1**). In fact, on the one hand, Ca-EVs activate NLRP3 inflammasome platform in non-immune receiving cells *via* ERK1/2-mediated pathway (12), on the other hand non-cancerous cell-derived EVs negatively modulate NLRP3 inflammasome activation in immune cells (13). This latter effect is mediated by EVs intrinsic metabolic activity that, through adenosine production, induces the activation of the adenosine A2a receptor, a member of the purinergic P1 receptor family (13). This novel mechanism of action highlights the active role of EVs in microenvironment homeostasis, *via* the autonomous synthesis of metabolic products able to alter microenvironment composition and cell behavior (13). Furthermore, the involvement of A2a receptor in this EVs effect, offers a novel point of view on the roles of EVs/purinergic receptors on cancer immunology (12). In fact, up to date, only the connection between EVs/type P2 purinergic receptors (P2Rs) and tumor-inflammatory signaling has been demonstrated (27). Only few reports demonstrate that the activation of P2Rs on immune cells induces the release of: (i) EVs containing IL-1β and IL-18, exerting a pro-inflammatory action, favor tumor progression at the expense of an effective immune response; (ii) EVs presenting P2Rs on their surfaces which activation, by extracellular ATP, can lead to the release of IL-1β, IL-18 and ATP itself (27). As discussed below, inflammasome and IL-1β dysregulation are crucial players in inflammation-associated tumorigenesis and chemoresistance. In this scenario, EVs by exerting an interaction-dependent effect on the receiving cells or by releasing immune-metabolites, can be considered novel crucial players in determining tumorigenesis and chemoresistance. The functional link between NLRP3 activation and EVs is further demonstrated by the finding that embryonic stem cell-derived EVs ameliorate the cardio-toxicity induced by the antineoplastic agent doxurobcin, by inhibiting NLRP3 signaling in mice (67). Nonetheless, further research is needed to increase the knowledge in this emerging research area.

## Inflammasome Targeting Drugs: Potential Anti-Cancer Therapeutic

Inflammation sustained by inflammasome activation has been implicated in the insurgence or progression of several human pathologies, including cancer. For this reason, several efforts have been made to identify potential effective inhibitors of inflammasome to be used as new anti-cancer therapeutics (summarized in **Table 1**). Each step leading to inflammasome activation, may represent a good candidate for therapeutic targeting.

Several small molecules and natural compounds have been identified as inhibitors of the interaction between NLRP3 inflammasome monomers. As examples MCC950 and OLT1177, block NLRP3 oligomerization by inhibiting ATP hydrolysis *via* the NACHT domain, which is pivotal for receptor oligomerization and anti-cancer effects (68). MC950 inhibits LPS-induced inflammasome activation in pancreatic cancer cell lines (69), delays cell growth in a mouse model of head and neck squamous cell carcinoma (70) and inhibits pituitary prolactinoma growth and prolactin expression/secretion in rats (71). Similarly, inhibition of NLRP3 by OLT1177 enhances antitumor immunity, thus reducing melanoma growth (68). Oridonin, a natural terpenoids found in traditional Chinese herbal medicine, impedes inflammasome assembly by forming covalent bond with NLRP3 Cys279 (72). Oridonin administration effectively prevents the formation of colorectal cancer liver metastasis (73) and improves oxaliplatin efficacy (74). Oridonin derivative, with potent anticancer effects, has been very recently synthesized (75).

ASC polymerization can be another target for broad-spectrum therapeutics. MM01 (under patent procedure: application number, 20382237.4-1109) is a small-molecule interfering with ASC speck formation (76). Xantone, used in the early twentieth century as an ovicide and larvicide (85) can inhibit ASC speck formation without affecting inflammasome components expression (77). Although MM01 and xantone can be useful for the treatment of a broad range of diseases based on inflammasome dysregulation, they have not yet been tested on cancer models.

Caspase-1 activation can be targeted for impeding IL-1β maturation. Caspase-1 inhibition by the small-molecule VX-765 prevents pyroptosis in a multiple sclerosis model (78) and in monocytes and macrophages (79). Ritonavir, originally used as protease inhibitor for the treatment of HIV, effectively block caspase-1 (80), and induces apoptosis in pancreatic cancer (81). However, the quite unspecific actions of protease inhibitors should be taken into account to avoid deleterious side effects.

Specific monoclonal antibodies directed toward IL-1 receptor, including anakinra, rilonacept, canakinumab and gevokizumab have been developed to inhibit IL-1β signaling (82). Anakinra, a recombinant IL-1Ra, blocking the binding of IL-1 to IL-1 receptor, is under clinical investigation for the treatment of metastatic cancers (ClinicalTrials.gov Identifier: NCT00072111). An up-to date list of clinical trials involving IL-1 blockade has been recently published (86). Nevertheless, the blockade of IL-1 receptor, although displaying a favourable safety profile, caused a reduction in neutrophil counts with an overall increased risk for fatal infections.

Besides the possibility to inhibit inflammasome components, several strategies aimed to inhibit the pathways leading to inflammasome activation. Antioxidant compounds can inhibit ROS-mediated inflammasome platform assembly, P2X7 receptor antagonist can be used to impede K^+^ efflux known to be involved in NLRP3 activation (86). A strategy, explored by our group, is the induction of NLRP3 phosphorylation. We have indeed showed that, natriuretic peptides (NPs), by binding to NPs Receptor-1, can induce an increase in cGMP levels which culminates in the activation of PKG (53, 83, 84). Moreover, we showed that EVs, isolated from amniotic fluid-derived stem cells can activate PKA *via* A2a adenosine receptor in immune cells (13). Both PKA and PKG can phosphorylate NLRP3 at Ser295, thus leading to the inhibition of inflammasome assembly and IL-1β secretion (13, 53). Furthermore, NPs are able to counteract both the constitutive and EVs-induced NLRP3 activation in cancerous and non-cancerous prostate cells (62), supporting the critical role of these molecules in prostate cancer (87). Based on the fact that NPs analogues are already in clinical use for cardiovascular diseases (88, 89) and of the growing interest toward the use of EVs as therapeutics (90), further studies are needed to better define the potential anti-cancer efficacy of NPs and EVs.

## Conclusion

Chemoresistance represents a major challenge in the clinic. Cancer cells response to therapy is deeply influenced by immune/inflammation-associated TME modifications. Therefore, the management of TME-mediated resistance may deeply affect the efficacy of cancer therapies. The key players that trigger TME modifications are multiple and strictly interconnected *via* a complex network of cell-cell communication. Given the pivotal role of inflammasome and related cytokines in TME re-modeling, they represent promising therapeutic targets for the development of novel anticancer approaches aimed to re-educate TME toward a favorable inflammatory/immune anti-tumorigenic phenotype. EVs have been recently discovered as novel active contributors of inflammasome/IL-1β modulation. Further studies are needed to better define the potential anti-cancer therapeutic efficacy of inflammasome-modulating drugs as adjuvant chemotherapy to counteract chemoresistance in a multidrug approach.

## Author Contributions

LM, IB, and RR contributed to conception of the study and wrote the manuscript. VT supervised the study. All authors listed contributed to manuscript revision, read, and approved the submitted version.

## Conflict of Interest

The authors declare that the research was conducted in the absence of any commercial or financial relationships that could be construed as a potential conflict of interest.

## Publisher’s Note

All claims expressed in this article are solely those of the authors and do not necessarily represent those of their affiliated organizations, or those of the publisher, the editors and the reviewers. Any product that may be evaluated in this article, or claim that may be made by its manufacturer, is not guaranteed or endorsed by the publisher.
